# Preparation and Properties of Double-Sided AgNWs/PVC/AgNWs Flexible Transparent Conductive Film by Dip-Coating Process

**DOI:** 10.1186/s11671-015-1022-0

**Published:** 2015-08-06

**Authors:** Cui-yu Chen, Mao-xiang Jing, Zhi-chao Pi, Sheng-wen Zhu, Xiang-qian Shen

**Affiliations:** Institute for Advanced Materials, Jiangsu University, Jiangsu, 212013 China

**Keywords:** Double-sided flexible transparent conductive film, AgNWs, Dip-coating, Figure of merit

## Abstract

The double-sided transparent conductive films of AgNWs/PVC/AgNWs using the silver nanowires and PVC substrate were fabricated by the dip-coating process followed by mechanical press treatment. The morphological and structural characteristics were investigated by scanning electron microscope (SEM) and atomic force microscope (AFM), the photoelectric properties and mechanical stability were measured by ultraviolet–visible spectroscopy (UV–vis) spectrophotometer, four-point probe technique, 3M sticky tape test, and cyclic bending test. The results indicate that the structure and photoelectric performances of the AgNWs films were mainly affected by the dipping and lifting speeds. At the optimized dipping speed of 50 mm/min and lifting speed of 100 mm/min, the AgNWs are evenly distributed on the surface of the PVC substrate, and the sheet resistance of AgNWs film on both sides of PVC is about 60 Ω/sq, and the optical transmittance is 84.55 % with the figure of merit value up to 35.8. The film treated with the 10 MPa pressure shows excellent adhesion and low surface roughness of 17.8 nm and maintains its conductivity with the sheet resistance change of 17 % over 10,000 cyclic bends.

## Background

Touch panels have a great market demand due to their brilliant operation performances [1–3]. As an electrode component for these touch panels, the double-sided transparent conductive film (TCF) with high transparency and conductivity on both sides of a substrate is demanded [4]. Currently, in the preparation process of double-sided transparent conductive films, two single-sided indium tin oxide (ITO) films were prepared firstly, and then the two single-sided ITO films were pasted on the two sides of polyethylene terephthalate (PET) substrate as upper or under circuit [5, 6]. This process is very complicated and prolixity. In addition, due to the high cost and fragility of ITO, there is a demand to replace ITO with other conductive films and develop a new preparation method for double-sided transparent conductive films.

There were transparent conductive films such as metal oxide film, polymer film and carbon nanotubes (CNT), graphene, and metal nanowire films (MNWs) [7–19]. Among these films, the MNWs film was attractive due to its high conductivity and transparency. In particular, the silver nanowires (AgNWs) films are promising for applications in optoelectronic devices resulting from their brilliant electrical, optical, and mechanical characteristics [20–22].

The AgNWs films were usually prepared by the Mayer rod coating [20, 22], vacuum filtration [21], and transfer-printing [23, 24], etc. Compared with these above processes, the dip-coating process [25–27] was convenient, low cost, and has been used to prepare for various films such as graphene-silver nanowires hybrid films [28] and carbon nanotubes films [29]. Especially, the dip-coating process is suitable to prepare double-sided coatings at the same time.

For the flexible transparent conductive films, the substrate is an important factor. Up to now, the AgNWs were usually deposited on a PET substrate. As the PVC and PET have a similar surface energy, contact angle and corrosion resistance, therefore, the PVC was used as the substrate for the double-sided AgNWs transparent conductive film in this work. The double-sided transparent conductive film was prepared by the dip-coating process, and the structure, photoelectric properties, and bending performance were also studied.

### Methods

The AgNWs suspension solution was bought from Coldstone Tech Co., Ltd (Suzhou, Jiangsu, China) with the concentration of 10 mg/mL. The diameter of the nanowires is approximately 70 nm with the length approximately 10~20 μm. The PVC substrate with thickness of 180 μm was washed with deionized water, acetone, or alcohol in ultrasonic vibration for 5 min.

**Fig. 1:**
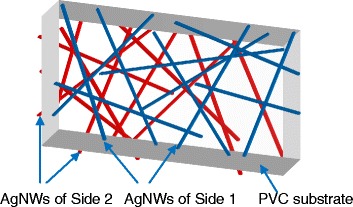
Schematic diagram of double-sided AgNWs/PVC/AgNWs transparent conductive film

The structure for the double-sided transparent conductive films of AgNWs/PVC/AgNWs was designed as shown in Fig. 1 and achieved by the following steps. First, the AgNWs suspension was diluted to a concentration of 1 mg/mL and supersonic vibrated for 10–30 s. The PVC film was vertically fixed on the dip arm and moved down into the AgNWs suspension at a dipping speed of 40~70 mm/min. When the PVC substrate was wholly dipped into the suspension and held for 30 s, the PVC film began to move up to the air at a lifting speed of 80~110 mm/min and air dried naturally for 30 s. This down and up process was repeated for three times according to a set program. Whereafter, the wet AgNWs/PVC films were carefully dried in a drying oven at 80 °C for 10 min to remove the organic solvent. To avoid agglomeration, heat treatment should be applied uniformly to avoid uneven local heating and damage to the substrate. In order to increase the adhesion properties, the films were mechanically pressed at 3 MPa and 10 MPa for 20 s successively by using a mechanical tablet machine with a pressure gage and a stopwatch [30]. The first press at 3 MPa is aimed to prepress the AgNWs for a low resistance, and the second press is to enhance the conjunction between AgNWs and with substrate.

The optical transmittance spectra were investigated by a Beijing PGeneral TU-1900 ultraviolet–visible spectroscopy (UV–vis) spectrophotometer (Beijing Purkinje General Instrument Co., Ltd., Beijing, China) with a blank substrate as the reference. The corresponding sheet resistance was measured using four-point probe technique at room temperature, and the average value was obtained from five measurements for each sample. The surface morphology of the films was observed by a JEOL JSM-7001 field emission scanning electron microscope (SEM; JEOL Ltd., Tokyo, Japan). The surface roughness of the AgNWs was investigated using a MicroNano D3000 atomic force microscopy (AFM; Shanghai Zhuolun MicroNano Instrument Co., Ltd., China). The adhesion performance was measured by 3 M adhesive tape test. The bending performance for the fabricated AgNWs films was determined by measuring the sheet resistance change of the films with the bending cycles at a bending radius of 5 mm, using a machine for reciprocating motion at a speed about 60 cycles/min. The sheet resistance was tested once every 1000 cycles.

## Results and Discussion

As shown in Fig. 1, the designed structure of AgNWs/PVC/AgNWs transparent conductive film includes two AgNWs films with the same structural property on both sides of PVC substrate, marked as Side 1 and Side 2. Actually, this structure was affected by many factors of dip-coating parameters, especially the dipping speed and lifting speed. The optical transmittance at *λ* = 550 nm (*T*_550_) and sheet resistance (*Rs*) of AgNWs/PVC/AgNWs films prepared in different dip-coating parameters are listed in Table 1. From Table 1, it can be found that the dipping speed and lifting speed are the important factors to influence the photoelectric properties of the AgNWs/PVC/AgNWs. Particularly, when the dipping speed is 40 mm/min and lifting speed is 90 or 100 mm/min, the sheet resistance of AgNWs film on both sides of PVC is about 45 Ω/sq or 51 Ω/sq and the transmittance is about 79.4 %.When the dipping speed is 50 mm/min and lifting speed is 100 mm/min, the sheet resistance of AgNWs film on both sides of PVC is about 60 Ω/sq and the transmittance reaches 84.55 %. This is the best compromise result for double-sided AgNWs/PVC/AgNWs film with high transmittance and low sheet resistance. In contrast, when the dipping speed and lifting speed is higher than 50 mm/min and 100 mm/min, respectively, most of the films have a sheet resistance over 200 Ω/sq with transmittance more than 85 %.

**Table 1:** Transmittance at *λ* = 550 nm and corresponding sheet resistance of AgNWs/PVC/AgNWs films with different dip-coating parameters

Sample no.	Dipping speed(mm/min)	Lifting speed(mm/min)	*T*_550_ (%)	*Rs* (Ω/sq) side 1	*Rs* (Ω/sq) side 2
1	40	80	83.44	172.6	172.8
2		90	79.42	45.0	45.4
3		100	79.46	51.0	51.2
4		110	81.38	159.1	159.2
5	50	80	85.17	121.4	121.7
6		90	84.25	94.6	94.3
7		100	84.55	60.1	60.4
8		110	85.24	133.6	133.4
9	60	100	85.12	197.0	197.2
10		110	85.96	201.2	201.5
11	70	100	87.12	246.0	246.3
12		110	88.58	284.1	284.3

**Fig. 2:**
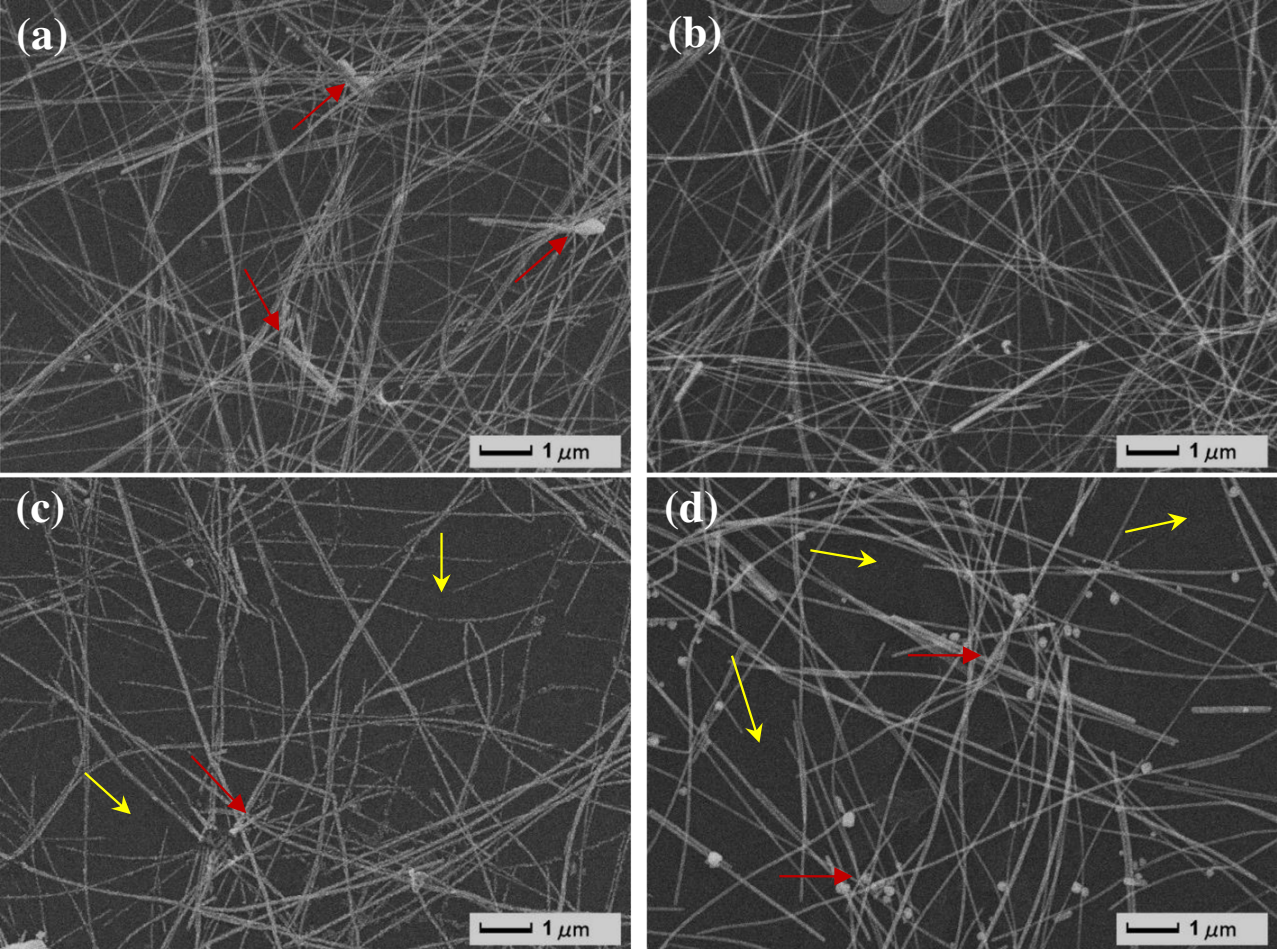
SEM morphologies of the AgNWs/PVC/AgNWs films prepared with different dipping speed and lifting speed by the dip-coating process: **a** dipping speed = 40 mm/min, lifting speed = 100 mm/min; **b** dipping speed = 50 mm/min, lifting speed = 100 mm/min; **c** dipping speed = 60 mm/min, lifting speed = 100 mm/min; **d** dipping speed = 70 mm/min, lifting speed = 100 mm/min

This photoelectric phenomenon of AgNWs network can be explained by the difference of their microstructure. Figure 2 presents the SEM images of AgNWs films (Side 1) fabricated by the dip-coating process with different dipping speed and lifting speed. From Fig. 2, it can be seen that the dipping speed and lifting speed also has great influence on the structure of the films. When the dipping speed is 40 mm/min and lifting speed is 100 mm/min, it shows that the PVC substrate is wholly covered with AgNWs, but in some areas, there appear some congestion of AgNWs as shown by red arrows in Fig. 2a. This microstructure can make the film have a low resistance but will influence the optical transmittance. While seen from Fig. 2b, the AgNWs film fabricated at a dipping speed of 50 mm/min and lifting speed of 100 mm/min clearly shows that the AgNWs are evenly distributed on the surface of PVC substrate without any congestion, which makes the film have high transmittance and low resistance at the same time. However, when the dipping speed increases to 60 mm/min or 70 mm/min and the lifting speed is 100 mm/min, the AgNWs are poorly distributed on the substrate, forming some congestion and open space as shown by yellow arrows in Fig. 2c, d, which may be caused by the fluctuation of the suspension when using too high speed. This open space can increase the light transmittance to some degree but conversely destroy the continuity of AgNWs network and result in the increase of resistance.

**Fig. 3:**
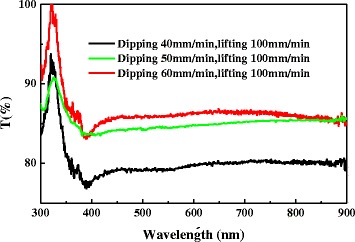
Optical transmittance spectra of AgNWs/PVC/AgNWs films fabricated with different dipping and lifting speeds

Figure 3 shows the transmittance curves of the AgNWs/PVC/AgNWs films over the range of wavelength 300–900 nm. From Fig. 3, the AgNWs/PVC/AgNWs film prepared at a dipping speed of 50 mm/min and lifting speed of 100 mm/min possesses a stable transmittance more than 84 % over a wavelength range of 400 to 900 nm, meaning that most visible light can transmit through this film, which is very important for the application of TCFs. While the films prepared at a dipping speed of 40 mm/min or 60 mm/min and lifting speed of 100 mm/min have a fluctuant transmittance that just agree with their microstructure as shown in Fig. 2a, c.

**Fig. 4:**
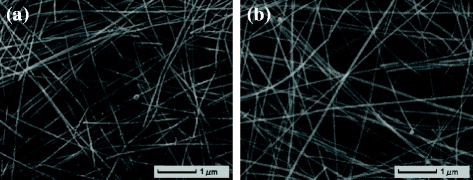
SEM morphologies of the AgNWs/PVC/AgNWs films prepared at the condition of dipping speed = 50 mm/min, lifting speed = 100 mm/min, **a** side 1 and **b** side 2

The SEM images for the AgNWs networks on both sides of the PVC substrate prepared at the dipping speed of 50 mm/min and lifting speed of 100 mm/min are shown in Fig. 4. From the SEM of side 1 and side 2, the AgNWs have formed a similar network and uniform distribution on the surface of PVC substrate. Therefore, using dip-coating process, the double-sided AgNWs/PVC/AgNWs with high transmittance and low resistance can be fabricated via one-pot step instead of pasting two single-sided conductive films on the two sides of the substrate, which greatly simplify the production process, cut down the cost, and increase the yield rate.

The TCF is generally required to have good electrical conductivity and high visible light transmittance. However, electrical conductivity and visible light transmittance are two contradictory parameters for a film. The figure of merit (FOM) is generally used to evaluate the performance of the transparent conducting films, which depends on both the transmittance and sheet resistance of a film. The ratio of DC conductivity and optical conductivity (*S*_dc_/*S*_op_) is calculated from the following equation [7, 31, 32]:

1 $$ FOM={Z}_0/2{R}_s\left({T}^{-1/2}-1\right) $$

**Fig. 5:**
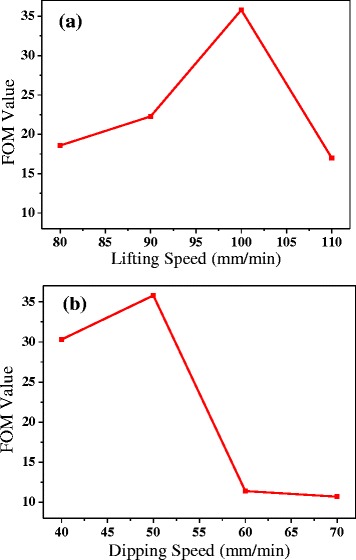
FOM value of **a** different lifting speed when the dipping speed is 50 mm/min and **b** different dipping speed when the lifting speed is 100 mm/min

In this equation, *Z*_o_ is the impedance of the free space and has the value of 377 Ω, *T* is the transmittance and *Rs* is the sheet resistance. From Eq. (1), we can notice that the FOM value will be high when the film with low sheet resistance and high transmittance. The transmittance and sheet resistance have illustrated that the dipping speed of 50 mm/min and the lifting speed of 100 mm/min is the best condition to form a uniform, high performance TCF. Figure 5 shows the FOM values of TCFs which prepared at different dipping speed or lifting speed. From Fig. 5a, b, when the dipping speed is 50 mm/min and lifting speed is 100 mm/min, the AgNWs film shows a high FOM value of 35.8. Some studies [7, 33] had pointed out that the minimum industry standard of FOM for replacing ITO material is 35. So the AgNW/PVC/AgNW film fabricated by the dip-coating method can meet the requirement of the minimum industry standard of FOM.

**Fig. 6:**
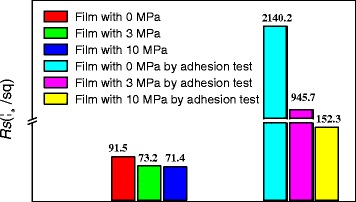
Change of sheet resistance of the films before/after adhesion test with 3 M adhesive tape

**Fig. 7:**
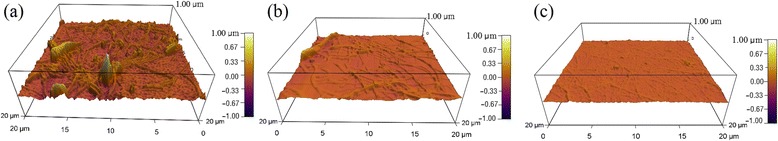
AFM images of AgNWs/PVC/AgNWs films treated with **a** 0 MPa, **b** 3 MPa, and **c** 10 MPa

Adhesion property between conductive film with substrate is a precondition for a film’s mechanical stability. From Fig. 6, it was clearly shown that the sheet resistance for the films with mechanical press at 3 MPa and 10 MPa changes from 73.2 Ω/sq to 945.7 Ω/sq and 71.4 Ω/sq to 152.3 Ω/sq, respectively, after tape testing. While the sheet resistance for the film without press increases from 91.5 Ω/sq to 2140.2 Ω/sq. In one respect, this result indicates that the adhesion between the substrate and the AgNWs film was significantly enhanced by mechanical press. On the other hand, the result also states that 3 MPa pressure for prepressing is just able to increase the conductivity of AgNWs film (*R*_*s*_ from 91.5 Ω/sq to 73.2 Ω/sq) by increasing the conjunctions between AgNWs but not enough to improve the adhesion between AgNWs and substrate. Therefore, 10 MPa pressure for second pressing is essential. This phenomenon is wholly consistent with our previous work [30]. The strong and stable adhesion might come from the film uniformity and tight junction between nanowires network and with substrate after second press treatment as shown in Fig. 7. From the AFM images of the films treated with 0, 3, and 10 MPa in Fig. 7, it can be clearly seen that the pressed film becomes smoother with the increase of pressure, the surface roughness of the films after press treatment of 0, 3, and 10 MPa is 42.5, 29.3, and 17.8 nm, respectively, and the AgNWs have been compacted tightly between each other and almost embedded into the substrate when pressed at 10 MPa.

**Fig. 8:**
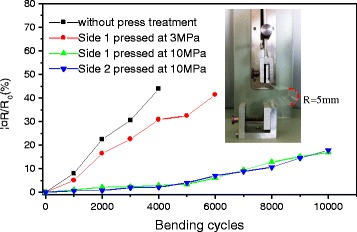
The change curves of sheet resistance with bending cycles. The inset picture shows the cyclic bending machine when bending, and the bending radius is 5 mm

To ensure the mechanical stability of the AgNWs/PVC/AgNWs films, the cyclic bending test was implemented as shown in Fig. 8. The inset picture shows the cyclic bending machine and the minimum bending radius is 5 mm. From Fig. 8, it can be seen that the sheet resistance change for the film without press treatment sharply goes up 43.8 % over 4000 cycles, the sheet resistance for the film with 3 MPa changes relatively slow with bending cycles but still rise by 41.3 % after 6000 cycles. While the film treated by 10 MPa pressing shows good bending performance with only 16.9 % up over 10,000 cyclic bends. These results coincide with the change of film’s structure and adhesion before and after press treatment. Notably, the sheet resistance of side 2 has the same change tendency as that of side 1 with 17.8 % change after 10,000 cycles, which indicates that the double-sided AgNWs/PVC/AgNWs film prepared by dip-coating process possesses a stable mechanical property.

## Conclusions

The double-sided transparent conductive films of AgNWs/PVC/AgNWs using AgNWs as conductor and PVC film as substrate were fabricated through facile dip-coating method at room temperature. The photoelectric properties of the films were greatly affected by the coating parameters. When the dipping speed is 50 mm/min and the lifting speed is 100 mm/min, the AgNWs film on both sides of PVC has a sheet resistance of about 60 Ω/sq with the optical transmittance of 84.55 %. The FOM value of AgNWs/PVC/AgNWs film is up to 35.8 that can meet the requirement of touch panels. The film treated with 10 MPa pressing possesses excellent adhesion and low surface roughness, which makes the film have high bending performance with about 17 % change of sheet resistance after 10,000 bending cycles.
